# Optimized grid representation of plant species richness in India—Utility of an existing national database in integrated ecological analysis

**DOI:** 10.1371/journal.pone.0173774

**Published:** 2017-03-15

**Authors:** Poonam Tripathi, Mukund Dev Behera, Partha Sarathi Roy

**Affiliations:** 1 Centre for Oceans, Rivers, Atmosphere and Land Sciences (CORAL), Indian Institute of Technology Kharagpur, Kharagpur, West Bengal, INDIA; 2 FNASc, FNAAS, NASI Senior Scientist Platinum Jubilee Fellow, Center for Earth & Space Sciences, University of Hyderabad, Andhra Pradesh, India; University of Delhi, INDIA

## Abstract

Data on the distribution of plant species at spatial (grid) scales are required as input for integrative analysis along with related climate, environment, topography and soil data. Although the world’s scientific community is increasingly generating data on plant species at various spatial grids and statistically interpolating and extrapolating the available information, data on plant diversity from the Asian continent are scant. Such data are unavailable for India, the mainland of which has part of three of the world’s 36 biodiversity hotspots. Although sufficient field sampling is always impossible and impractical, it is essential to utilize fully any available database by adjudging the sampling sufficiency at a given scale. In this work, we used an exhaustive database of the plant species of the Indian mainland that was sufficient in terms of sampling vegetation types. We transformed the data, obtained the distribution at the 1° and 2° spatial grid levels and evaluated the sampling sufficiency at acceptable threshold limits (60% to 80%). The greatest species richness values recorded in the 0.04 ha quadrant, 1° grid and 2° grid were 59, 623 and 1244, respectively.

Clench model was significantly (p value < 0.001) fitted using the plant species data at both the grid levels with a very high coefficient of determination (>0.95). At an acceptable threshold limit of 70%, almost all the grids at the 2° level and more than 80% of the grids at the 1° level were found to be sufficiently sampled. Sampling sufficiency was observed to be highly scale-dependent as a greater number of 2° grids attained asymptotic behaviour following the species–area curve. Grid-level sampling insufficiency was attributed to lower numbers of sampling quadrats in forests with poor approachability, which coincided with the world biodiversity hotspots’, suggesting that additional sampling was required. We prescribe the use of the 1° and 2° spatial grids with sufficient sampling for any ecological analysis in conjunction with other data and thereby offer grid-level plant species richness data for the Indian mainland for the first time.

## Introduction

The heterogeneous distribution of species across biomes and concerns for their continued existence in the future inspire ecologists and bio-geographers. The interest in understanding them lies in improving the available data set and analytical tools through taxonomic inventory, collation of the existing specimens and using remote sensing data for monitoring vegetation [1]. Plant diversity data analysis has always had constraints at all scales from the local to the global [2]. This problem can be resolved by transforming scattered data to an appropriate spatial level or scale so that they can be uses with other collateral data sets for integrated analysis.

It is imperative to monitor and map vegetation through taxonomic inventories with sufficient sampling [3]. Incomplete sampling and biases may lead to erroneous interpretation of ecological facts, affecting conservation and policy implications [4]. The common biases might be introduced in inventories: (i) species (more sampling of one species relative to others); (ii) species–area (over- or under-sampling of a species in relation to the size of the area); (iii) hotspot (excessive or insufficient collection in certain geographic areas); and (iv) infrastructure bias (proximity to roads and residence) [5]. The sampling bias is also related to the spatial scale. For example, at coarser scales, sampling biases get weaker due to the improved data coverage. Nevertheless, it is impossible to conduct complete, uninterrupted numerical inventories at regular intervals due to various limitations including site accessibility, cost-effectiveness and time [6], [7]. Therefore, it is essential to make full use of any available database by judging the sampling adequacy at a given scale even if the data had been collected with any pre-defined objectives [8]. In general a sampling sufficiency value between 70% and 90% is often considered to be acceptable and fit for any further ecological analysis [9].

Various statistical models have been used in the past to assess sampling sufficiency: (i) log–normal distribution fitting to species abundance data, (ii) asymptotic curve fitting to species accumulation curves and (iii) using non-parametric estimators based on the abundance or incidence of rare species [10],[11]. The most common non-parametric estimators are the Chao [12], jackknife and bootstrap [13] estimators. These predict the species richness on the basis of the number of rare species observed within samples, either from incidence data or from abundance data. Species accumulation models have been successfully fitted to various taxonomic groups including animals and plants [14], [15]. The accumulation curves obtained (asymptotes showing the cumulative number of species against sampling effort) are useful in describing the rates of addition of new species to inventories [16], [17]. These curves attain asymptoteness when the probability of addition of new species approaches zero; they are non-asymptotic otherwise. Two parameters have been suggested for assessing sampling sufficiency: (i) the slope of the species accumulation curve, which describes the rate of addition of new species [18], [8], and (ii) the ratio of the total species richness expected from the richness estimators to the observed species richness [19], [20]. Recently Pardo et al. [14] used FIDEGAM, which is based on species accumulation curves, under different scenarios of sampling exhaustiveness, with receiver operating characteristic (ROC) analyses to quantify sampling sufficiency. The use of various techniques involving repeated sampling and accumulation curves to estimate species richness has been explored [21], [22]. The species richness in a controlled area, *i*.*e*., grid area, depends not only on the inventoried plots but also on the area of the vegetation. For example, we may expect a smaller number of species in a grid that falls within an area that is having less than 1% of the vegetation area even if the grid covers 100% of the geographic area. This is due to the area restriction, which constrains an investigator from laying more plots because of the lack of vegetation. Therefore, the proportion of the vegetation area might serve as a criterion in assessing sampling sufficiency in asymptotic models.

Clench equation was fitted to obtain species accumulation curves (e.g., Jiménez-Valverde et al., [23]) to assess the sampling sufficiency of the species inventory of each grid. Clench function performs better by avoiding the problem of over-fitting and under-estimation of the critical richness and, therefore, is the most widely used function among asymptotic estimators [24]. We examined the increase in the number of species with the addition of sampling areas in a grid.

Plant species inventories carried out in India in the past are discrete—there are variations at the local/regional level and in the size of the quadrat. Thus they fall short of the requirements of country-level representation for interdisciplinary and multidisciplinary studies. The plant database generated during the execution of a national-level project, ‘Biodiversity Characterization at Landscape Level’, was utilized in this study to overcome this limitation. The data were collected in 0.04 hectare for tree species. Nested quadrats were used with a stratified random sampling approach [25]. To sample shrub species, two plots of size 5 m × 5 m each were laid at opposite corners of tree plots, and for herb species five plots of size 1 m × 1 m each were laid at the four corners and centre of tree plots. A 1:50,000 scale vegetation type map was generated using satellite remote sensing data and an on-screen visual interpretation technique [26]. The map had 100 vegetation classes wherein, the accuracy of the map was verified using the information obtained by sampling and was found to be greater than 90%. The vegetation type and area greatly affect the number of species. Spatial knowledge of these variables with respect to species number will help understand the pattern. An analysis of the sampling sufficiency will be useful in identifying grids in which sampling is insufficient. More effective survey campaigns can be designed in the future. In the present work, we have provided for the first time the pattern of plant species richness distribution in the Indian mainland in 1° and 2° grids. The main objectives of our study were (i) to analyse the sampling sufficiency at both the grid levels (1° and 2°) at various sufficiency thresholds (60% to 80% at 5% intervals) and (ii) to propose priority-basis grid sampling in the under-sampled regions.

## Methods

### Study area

The present work was carried out for the Indian mainland (S1 Fig). India, a large country with a total geographical area of nearly 329 million hectares, lies to the north of the equator, between latitudes 6° 44ʹ N and 35° 30ʹ N and longitudes 68° 7ʹ E and 97° 25ʹ E. India has a wide range of bio-climatic zones with distinctive ecology, biomes, communities and species [27]. The climate of the country varies from temperate in the north to monsoonal in the south. Four major monsoonal seasons are recognizable in the country: (i) essentially warm and humid south-west (SW) summer monsoon, from June to September [28], (ii) north-east winter monsoon, from October to December [28], (iii) cold and dry winter monsoon, from December to February [29], and (iv) spring, from March to May [30]. Most of the annual rainfall (80%) is received during the SW summer monsoon. The large spatial variability of the monsoonal activity has accounted for the diverse eco-regions and consequently the vegetation types in the country. The Himalayan orography/topography plays an important role in defining variations in precipitation patterns of the mountain ranges [31]. This supports heterogeneous vegetation types including sub-tropical, temperate, alpine forms alpine pastures and scrub in Himalayan regions. The heavy rainfall of south-western and north-eastern India provides favourable conditions for evergreen and moist deciduous forests. Being climatically diverse, the Himalayan (East and West Himalaya) and south-western regions (Western Ghats) support high species richness and therefore have been included among the major hotspots of the world [32]. The western and north-western regions, with low annual precipitation, support desert and semi-arid ecosystems, and therefore mostly thorn forests with low species richness are prominent.

### Data source

We used the species richness data collected from the national-level project ‘Biodiversity Characterization at Landscape Level’ [25]. The analysis was performed at two grid levels, namely, 1° and 2°. Fishnets of two grid sizes covering the whole study area were created using the ‘Create Fishnet’ tool of ArcGIS 9.3. The counts of unique plant species were spatially appended to the respective grid cells using the spatial join method. The two grid levels were overlaid on each other such that four grids of the 1° level contributed to a 2° grid level, leading to an increased grid area (Fig 2) and subsequently more sample points. Finally, plant species raster data at two scales (1° and 2°) were generated using the ‘Polygon to Raster’ tool of ArcGIS 9.3 (S2 Fig). We utilized the vegetation type map of India [26] to extract the vegetation area information at each grid cell using the ‘Zonal Statistics’ tool (S2 and S3 Tables). All the maps were generated using the ArcMap 9.3 software package.

### Species richness estimation and sampling sufficiency

We fitted Clench equation to obtain the species accumulation curves to assess the degree of sufficiency of the species inventories in each grid [23]. Therefore, we examined the increase in the number of species accumulated with additional sampling in each grid. The curve suggests that the probability of adding new species to the list decreases as the number of species already recorded increases, but it increases over time. The curve follows a convex function until an ideal asymptoteness of maximum species is attained, where the survey effort is almost infinite [33]. The equation is

S(x)=ax/(1+bx), (1)

where, x is a measure of the sampling effort, S(x) is the predicted number of species at effort x, a represents the rate of increase at the beginning of the sampling, b is related to the mode of accumulation of new species during sampling and a/b indicates asymptoteness.

The models were fitted using the Gauss–Newton method for least square non-linear problems using Statistica 7.0. The sampling sufficiency values were calculated using the asymptotic value of Clench function for the cumulative number of species records [33]. The ratio of the observed species richness to the asymptotic predicted species richness was calculated as a measure of the grid sufficiency. Clench function was fitted only to 277 and 92 grids at the 1° and 2° levels, respectively. Clench function was chosen because its fitness allows the estimation of higher asymptotic values and thereby demonstrates more exigent degrees of sufficiency [34].

We used five sufficiency thresholds with respect to ±70% at 5% of variations as 60%, 65%, 70%, 75% and 80% of total Clench predicted values. We also calculated the theoretical effort required (n_q_, in this case n_0.70_) to reach 70% of each inventory, which, according to Soberón and Llorente [33], is given as

$$n_{q}=q/\left[b\left(1\;\text{-}\;q\right)\right],$$ (2)

where q is the relative proportion of the list of species to be detected.

We calculated the overall grid area, occupied Indian geographic area and vegetation area within each 1° and 2° grid using the ‘Zonal Statistics’ tool in ArcGIS 9.3.

We proposed to lay out the quadrats at the 1° grid level on a priority basis in the under-sampled grids using the heterogeneity and vegetation area as the criteria. For the purpose, we extracted the vegetation type (a measure of heterogeneity) and grid vegetation area of each grid. The weighted method was used to define the sampling priority of each grid. Higher weights were assigned to the grids having (i) the maximum grid vegetation area occupancy and (ii) the maximum number of vegetation types. We used a 0–100 scale to measure both criteria (i.e., vegetation type and vegetation area), with 0 being the least desired outcome for each criterion and 100 the most desired outcome. Among the under-sampled grids, in assigning weights for vegetation type, 1 was the least desired outcome and 13 was the most desired outcome. For example, a grid having the maximum number of vegetation types (13) will get a score of 100%:

[(13-1)/(13-1)=1and1×100=100%].

Similarly, a grid having only one vegetation type will have a score of 0:

[(1-1)/(13-1)=0].

The weightage for the vegetation area was defined similarly.

We assumed both the criteria to be equally important for sampling, and therefore they were each assigned 50% weightages. Further, their weighted sum was calculated to define the priority grids for additional sampling.

## Results

### Species richness pattern

A total of 2,87,675 records for 6019 unique species from the Indian mainland were gathered from 15,529 nested quadrats distributed over 100 vegetation types (S2A Fig). The greatest number of quadrats (9158) was observed in the mixed forest class, with 20 vegetation types, mostly tropical evergreen, sub-tropical broad-leaved, Himalayan temperate, sub-alpine, tropical sal and teak mixed deciduous, and temperate coniferous forests (refer to Roy et al. [26] for all vegetation types). The smallest number of quadrats (161) was in woodlands, with only three vegetation types: woodlands, tree savannah and shrub savannah (S1 Table).

Out of a total of 301 grids at the 1° level and 95 grids at the 2° level in the Indian mainland, 24 and three grids were observed to have fewer than five plots, respectively. These were discarded from further analysis. A total of 207 and 40 grids were fully covered within the Indian geographic area, leaving 94 and 55 grids to be partially covered at the 1° and 2° grid levels, respectively (S2 and S3 Tables). The cumulative number of species was enumerated for all grids falling within the Indian mainland that had forest vegetation (S2B and S2C Fig). The pattern of species richness varied geographically. The number of species varied from a minimum of 1 to a maximum of 59 at the individual quadrat level (S2A Fig). The ranges of species richness had minima of 3 and 6 and maxima of 623 and 1244 at the 1° and 2° grid levels, respectively (S2B and S2C Fig). The highest number of species at the quadrat level (59) was found in the south-eastern region of India, while most of the lower numbers of species, ranging from 1 to 8, were found in western India. At the 1° grid level, the greatest number of species (623) was observed in the transition zone of Sikkim and West Bengal in the Himalayan region. At the 2° grid level, the greatest number of species (1244) shifted spatially to the southern region, including the Western Ghats. The lowest numbers of species (3 and 17) were observed in the western region, corresponding to Gujarat. There was no spatial shift at both the grid levels. At the 1° grid level, the maximum vegetation area (92.78%) was occupied in grids corresponding to north-eastern Arunachal Pradesh, and nearly 45 grids had vegetation area greater than 50% (S2 Table). At the 2° grid level, only seven grids had vegetation area greater than 50% and 14 grids had vegetation area less than 5%. The maximum vegetation area (79.76%) corresponds to the grids falling in north-eastern India, including Assam, Meghalaya, Nagaland, Manipur, Mizoram and Tripura; the least vegetation area (0.31%) was in a 1° grid falling in Odisha (S3 Table).

### Sampling sufficiency

Clench model fitness curves for all grids showed variations in the number of sufficiently sampled grids at various thresholds, with sampling sufficiency inversely proportional to the threshold limits. Clench model fitted well at both grid levels (r^2^ > 0.95 and p value < 0.001); however, it tended to over-predict the true number of species by estimating higher degrees of asymptoteness (S2 and S3 Tables). As the threshold of the sampling sufficiency increased at 5% steps, the number of grids meeting the criteria decreased. Among 277 and 92 grids at the 1° and 2° levels, a total of 254 and 90 grids were observed to be sufficiently sampled at the 60% threshold level, respectively (Table 1). Total numbers of grids of 243 and 88; 219 and 85; 177 and 77; and 119 and 66 were found to be sufficiently sampled at the 65%, 70%, 75% and 80% threshold levels for the 1° and 2° grids, respectively (Table 1).

**Table 1:** number of grids at different threshold level showing sampling in-/ sufficiency in Indian mainland.

	1 degree			2 degree		
% Threshold level	Sufficient		Insufficient	Sufficient		Insufficient
	Excluding <5% veg area	Including <5% veg area		Excluding <5% veg area	Including <5% veg area	
60	254 (91.70)	261 (94.22)	23 (8.30)	90 (97.83)	91 (98.91)	2 (2.17)
65	243 (87.73)	254 (91.70)	34 (12.27)	88 (95.65)	90 (97.83)	4 (4.34)
70	219 (79.06)	231 (83.39)	58 (20.93)	85 (92.39)	89 (96.74)	7 (7.60)
75	177 (63.90)	195 (70.40)	100 (36.10)	77 (83.70)	84 (91.30)	15 (16.30)
80	119 (42.96)	144 (51.99)	158 (57.03)	66 (71.74)	76 (82.61)	26 (28.26)

* In parentheses % of sampling.

The number of sufficiently sampled grids decreased at higher threshold limits. The maximum numbers of grids with under-sampling at the 60% threshold limit were 91.7% and 97.8% for the 1° and 2° levels, respectively, when grids with vegetation area less than 5% were not considered. At the 80% threshold limit, 42.9% and 71.7% of the grids were found to be under-sampled at the 1° and 2° levels, respectively. The numbers of grids with vegetation area less than 5% at various threshold limits for the 1° and 2° grid levels were found to be 7 and 1, 11 and 2, 12 and 4, 18 and 7, and 25 and 10, respectively. Therefore, the number of sufficiently sampled grids increased when the <5% vegetation area criterion was implemented, thus including a few more grids (Table 1). At a threshold limit of 70%, more than 80% of grids were found to be sufficiently sampled. When the <5% vegetation area criterion was considered, nearly 84% and 90% of the grids were sufficiently sampled at the 1° and 2° levels with a 70% threshold limit (Table 1).

**Fig 1:**
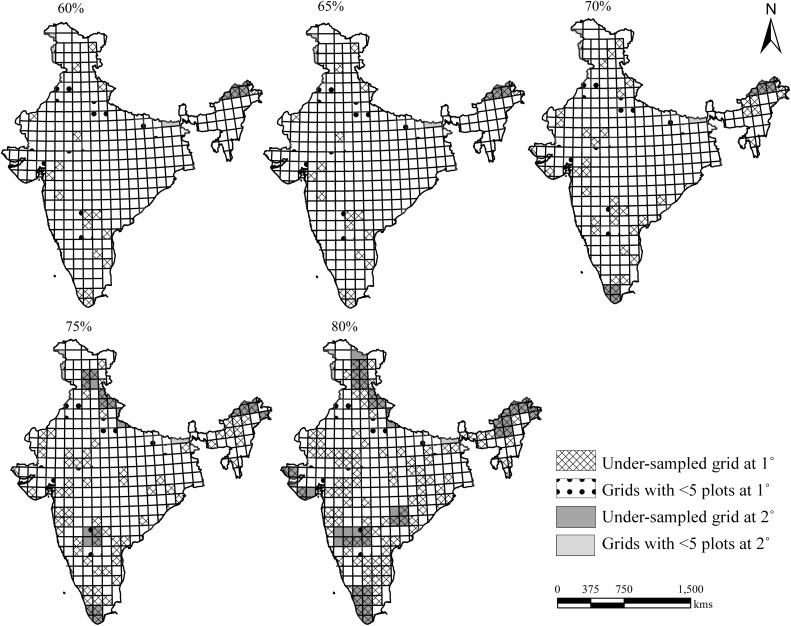
Spatial representation of sampling: Insufficiently sampled grids at various threshold levels (mentioned on top) for 1° and 2° grids; it may be noted that all the grids those remained insufficiently sampled at 2° are bound to remain insufficiently sampled at 1° [*1° grids are shown under the backdrop of 2° grid].

Most of the insufficiently sampled grids were in the hotspot regions of India at both grid levels. At the 1° grid level, for the 80% threshold limit, most of the under-sampled grids were in the forest-rich regions of the Himalaya, the North-east, the Western Ghats and the arid regions, including the Eastern Ghats in the Deccan Peninsula Zone (Fig 1). At higher threshold limits, there were more under-sampled grids in the Himalaya, the Western Ghats and the arid zones at the 2° grid level. The sampling sufficiency was greater in the Gangetic plain (including the major part of the state of Uttar Pradesh) and the arid regions. The theoretical number of samples needed to reach grid-level inventories at 70% (N70%) for the under-sampled grids varied from 6 to 137 plots and from 22 to 94 plots for the 1° and 2° grid levels, respectively (S2 and S3 Tables).

**Fig 2:**
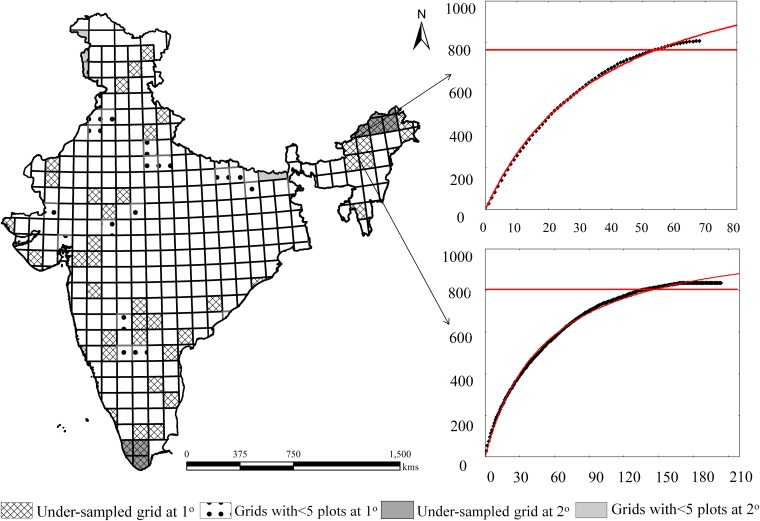
Clench fitted species accumulation curves for (a) 1° grid and (b) 2° grid (attaining asymptoteness); *Highlighted in black color shows grid at 2° scale (a and b values shows no. of observed species, no. of asymptotic species (in brackets) and; total no. of sampling plots for the particular grid).

## Discussion

The sampling effort of the project ‘Biodiversity Characterization at Landscape Level’ demonstrates sampling sufficiency with a 70% threshold limit at the 1° and 2° grid levels for the Indian mainland. In general, a sampled species, when reaches at least 70% of the estimated species for the particular group is considered the representative of the existing species richness [35], [36].

### 1° grid

The sufficiency of grids is mostly due to a greater number of sampling plots, more infrastructure and better road access to forests. Most of the insufficiently sampled grids correspond to the Himalaya and the Trans-Himalayan Zone, in north and north-eastern India. This might be explained in terms of accessibility. The region spans an elevation range from below 100 m to above 5000 m. It has the maximum deviation (nearly 1000 m) at 1°, and the average temperature is ca. 8–10°C. The harsh climate limits the accessibility of the region to the investigator for sampling. Most of the studies have highlighted the issue of infrastructure biases, with sampling being mostly influenced by the road network and infrastructure, leading to insufficiently sampled areas [5]. Grids in the Western Ghats and parts of the Deccan Peninsula Zone might be under-sampled due to the undulating terrain and restriction of accessibility. In contrast, most of the sufficiently sampled grids in the Gangetic plains and parts of the Deccan Peninsula are in close proximity to accessible areas.

### 2° grid

In the 2° grid, the species richness increases due to the cumulative effect of area. The curve is more asymptotic in the 2° grid than in the 1° grid, essentially showing higher sufficiency (Fig 2). Sampling is highly dependent on the vegetation area. This is because high vegetation area in a limited region (the grid in this case) offers investigators to lay more plots and vice versa. The under-sampled grids at the 2° grid level (75% and 80% thresholds) in the Western Ghats and the lower Deccan Peninsula were observed because of the presence of <5 quadrats at the 1° grid level.

Sampling sufficiency was observed to be highly scale–dependent, with greater sufficiency at coarse grid level (Table 1). This might be due to the probability of larger numbers of sample plots in coarse grids, which increases homogenous vegetation type and more common species and supports the species–area curve hypothesis. Hence, the use of asymptotic functions facilitates rigorous comparisons by assessing the sampling sufficiency as demonstrated in the present study [37], [33]. Clench model gives an estimate of the true number of species’ with the prediction being at the ‘upper-limit’ asymptote. This is its strength in predicting species in species-rich regions, i.e., hotspots. However, this can be reversed in species-poor areas, where the model predicts a larger number of species where the number is actually low.

The survey was conducted specifically for the purpose of completing vegetation type–wise sampling. Therefore the investigator might have spent more effort in laying plots in regions with a specific vegetation type, ignoring under-sampled regions. We found a few densely sampled grids in our data, with high species richness, overlapping hotspots. These can be regarded as true hotspots, and thus the observed patterns can be regarded as valid in spite of the unequal sampling. More rare and endemic species can be found in hotspots, and so with additional sampling, the species richness will increase.

### Prescriptions for additional sampling

**Fig 3:**
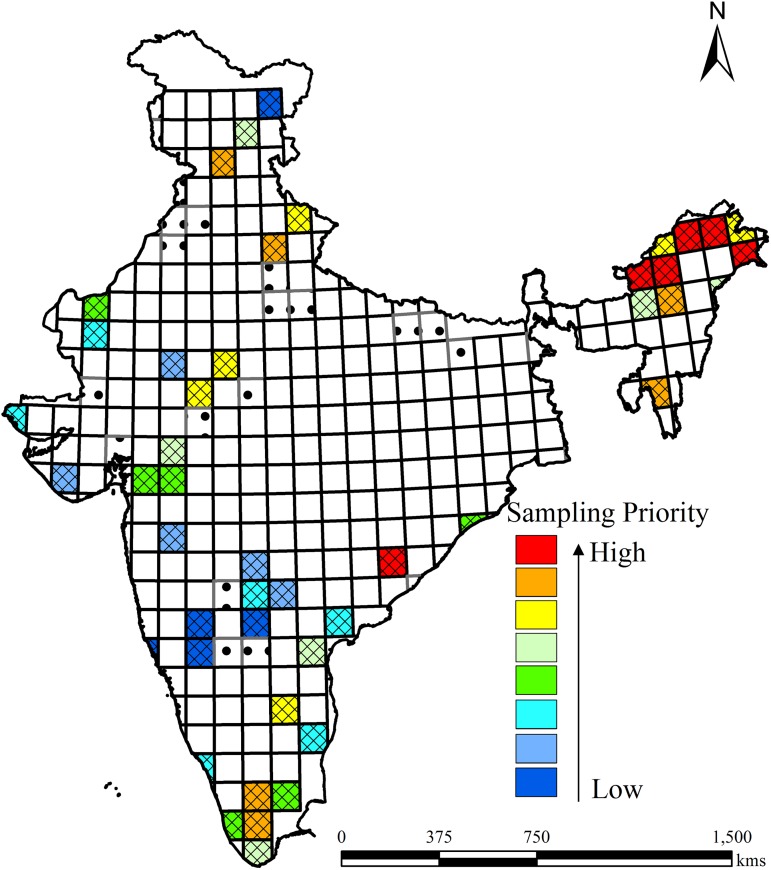
Spatial representation of insufficiently-sampled grids showing sampling priority based on the cumulative measures of heterogeneity and area. Red indicates high priority grids and blue indicates low priority grids for additional sampling.

We propose to lay out quadrats at the 1° grid level on priority basis in the under-sampled grids (46; 16.6%) using the criteria of heterogeneity and vegetation area (Fig 3). Heterogeneity plays a major role in determining the species richness pattern. In an area with heterogeneous vegetation types we can expect a greater number of species due to the distinct environmental conditions. We observed that the grids from the Himalayan zone in north-eastern India suffer severely from under-sampling and need to be urgently sampled since this is a major hotspot of India. The priority grids clearly show that the under-sampled grids are mostly in hotspots regions and require more sampling (Fig 3).

Although choosing a coarser grid level improves the sampling sufficiency, opportunities for interpretation and ecological applications are poorer. The findings of the present study highlight a significant issue of conserving plant biodiversity since coarse grid-level data are built upon species inventoried at fine grid levels. For conservation research, the issue is critical because fine grid-level studies are commonly used as the basic platform to estimate extinction risks of species [38].

A spatial database is the need of the hour, and therefore the sampling adequacy of more than 75% of the grids at the 1° and 2° grid levels represents the strength of our species database, which can facilitate various researchers and scientific communities working at levels ranging from the regional to the global.

This database will not only be helpful in linking and harmonizing various themes of raster data, i.e., environmental data, so that they can be used as input in multi-scale models, but will also be useful in aggregating data at multiple resolutions and using them in relational database management systems.

## Conclusions

Assessment of sampling sufficiency is crucial for any comprehensive study of plant species richness patterns. Adequately sampled areas can be used to assess absence of species to improve models of predicting species distribution patterns. Our study clearly reveals the sampling-sufficient and sampling-deficient regions of the Indian mainland at the 1° and 2° grid levels with various threshold limits. As expected, at lower sufficiency thresholds, larger numbers of grids were found to be sufficiently sampled. Larger numbers of grids were sufficiently sampled at the 2° grid level compared with the 1° level. The addition of environmental variables might help obtain more comprehensive pictures of broad-scale patterns of species. Field surveys should be carried out in regions that are found to be under-sampled. Population density and infrastructure are surrogate variables for predicting sampling sufficiency. Our results may serve as a guiding tool for future sampling efforts to generate a grid-level plant diversity database of India.

## Supporting information

S1 Fig Study region showing Indian state boundaries and biogeographic zones (as per Rodgers and Panwar [26] overlaid. (DOCX)

S2 Fig Species distribution in Indian mainland **a.** Number of species enumerated in a 0.04 ha nested quadrats (Maximum is 59); **b.** Represented at 1^0^ x 1^0^ grids (maximum is 623); **c.** Represented at 2^0^ x 2^0^ grids (maximum is 1244).
(DOCX)

S1 Table Major vegetation classes has been grouped under 9 from the 100- types as mapped by Roy et al. (2015); the number of quadrates laid for plant species inventory is also mentioned. (DOCX)

S2 Table Table showing the observed and asymptote species richness of India at 1° scale. (DOCX)

S3 Table Table showing the observed and asymptote species richness of India at 1° scale. (DOCX)
