# Supplementary material for: Optimized grid representation of plant species richness in India—Utility of an existing national database in integrated ecological analysis
Source: PLoS One. 2017 Mar 15;12(3):e0173774. doi: 10.1371/journal.pone.0173774 (PMC5352167; doi:10.1371/journal.pone.0173774)
Supplement: S1 Fig — (DOCX) [file pone.0173774.s001.docx]

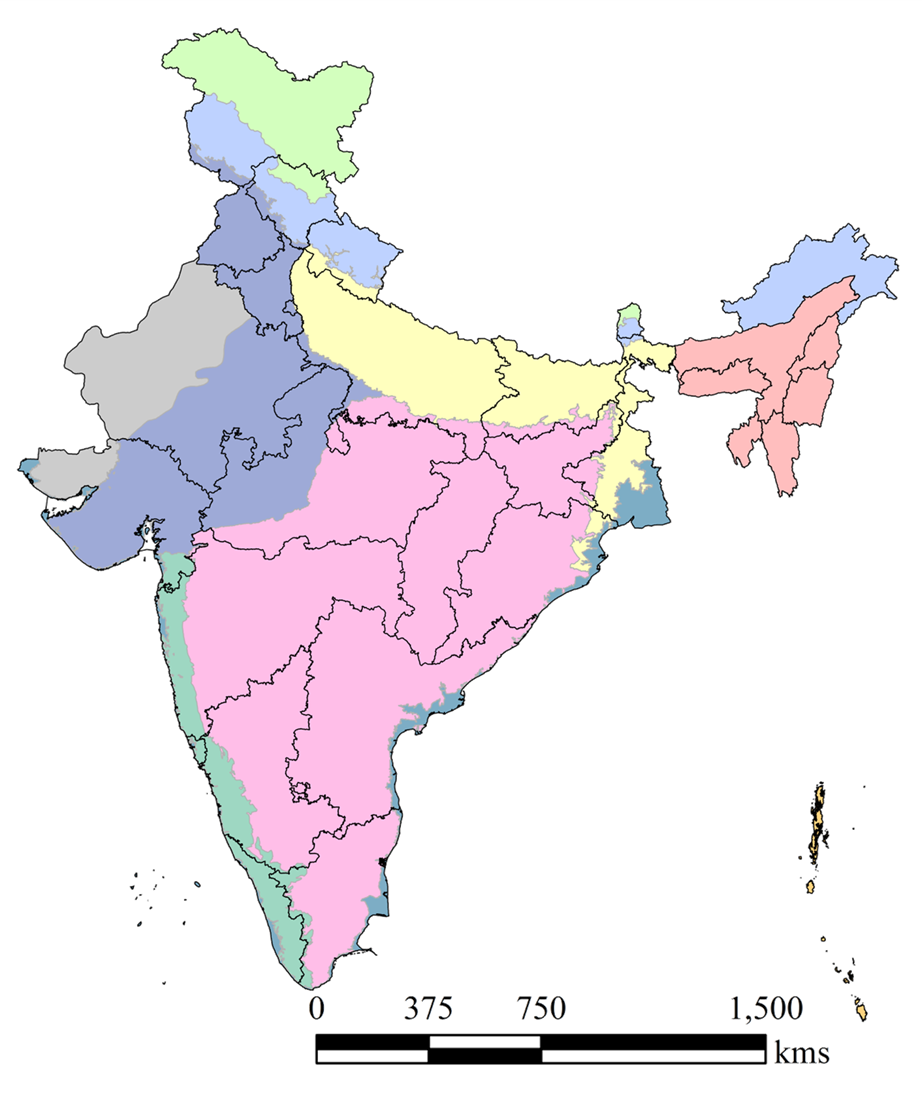


Uttar Pradesh

Madhya

Pradesh

Arunachal

Pradesh

Assam

Odisha

Andhra

Pradesh

Tamil Nadu

Maharashtra

Karnataka

Kerala

Chhattisgarh

Jharkhand

West Bengal

Rajasthan

Gujarat

Uttarakhand

Haryana

Punjab

Jammu & Kashmir

Himanchal Pradesh

Bihar

Sikkim

Meghalaya

Nagaland

Manipur

Mizoram

Tripura

Goa

Lakshadweep

Andaman & Nicobar

**S1 Fig. Study region showing Indian state boundaries and biogeographic zones (as per Rodgers and Panwar [26] overlaid**
