# Supplementary material for: Optimized grid representation of plant species richness in India—Utility of an existing national database in integrated ecological analysis
Source: PLoS One. 2017 Mar 15;12(3):e0173774. doi: 10.1371/journal.pone.0173774 (PMC5352167; doi:10.1371/journal.pone.0173774)
Supplement: S2 Fig — Species distribution in Indian mainland a. Number of species enumerated in a 0.04 ha nested quadrats (Maximum is 59); b. Represented at 10 x 10 grids (maximum is 623); c. Represented at 20 x 20 grids (maximum is 1244). (DOCX) [file pone.0173774.s002.docx]

1. **b. c.**

124-250

250-373

373-498

498-623

623-746

746-871

871-995

995-1120

1120-1244

1- 6

6 -12

12-18

18 -24

24-30

30-36

36-42

42-48

48-54

54-59

**Plot SR Grid**

0-124

**S2 Fig. Species distribution in Indian mainland a. Number of species enumerated in a 0.04 ha nested quadrats (Maximum is 59);**

**b. Represented at 1^0^ x 1^0^ grids (maximum is 623); c. Represented at 2^0^ x 2^0^ grids (maximum is 1244)**
