# Supplementary material for: Optimized grid representation of plant species richness in India—Utility of an existing national database in integrated ecological analysis
Source: PLoS One. 2017 Mar 15;12(3):e0173774. doi: 10.1371/journal.pone.0173774 (PMC5352167; doi:10.1371/journal.pone.0173774)
Supplement: S1 Table — (DOCX) [file pone.0173774.s003.docx]

**S1 Table. Major vegetation classes has been grouped under 9 from the 100- types as mapped by Roy et al. (2015); the number of quadrates laid for plant species inventory is also mentioned**

| **Major vegetation class** | **Plots laid** | **Number of vegetation type** | |
| --- | --- | --- | --- |
| Degraded forest | 683 | 4 |  |
| Grassland | 1079 | 11 |  |
| Gregarious forest | 1714 | 17 |  |
| Locale specific | 338 | 17 |  |
| Managed forest | 175 | 8 |  |
| Mixed forest | 9158 | 20 |  |
| Plantation | 225 | 7 |  |
| Scrub/Shrub land | 1996 | 13 |  |
| Woodland | 161 | 3 |  |
