# Supplementary material for: Optimized grid representation of plant species richness in India—Utility of an existing national database in integrated ecological analysis
Source: PLoS One. 2017 Mar 15;12(3):e0173774. doi: 10.1371/journal.pone.0173774 (PMC5352167; doi:10.1371/journal.pone.0173774)
Supplement: S2 Table — (DOCX) [file pone.0173774.s004.docx]

**S2 Table. Table showing the observed and asymptote species richness of India at 1˚ scale**

| S. no. | Observed SR | Plot | Total grid area (km^2^) | Indian geo. area (km^2^) | Indian veg area (km^2^) | Total grid veg area (%) | Asymptote (a/b) SR | Expected effort to 70% completeness (N70%) |
| --- | --- | --- | --- | --- | --- | --- | --- | --- |
| 1 | 18 | 1* | 10184.35 | 3907.09 | 3504.61 | 34.41 | ----- | ----- |
| 2 | 136 | 70 | 10180.12 | 10106.1 | 6079.08 | 59.72 | 158.53 | 18 |
| 3 | 238 | 93 | 10180.12 | 10180.12 | 5236.04 | 51.43 | 294.73 | 38 |
| 4 | 267 | 66 | 10184.35 | 10184.35 | 3392.21 | 33.31 | 349.05 | 38 |
| 5 | 234 | 50 | 10192.82 | 10192.82 | 2022.49 | 19.84 | 308.75 | 32 |
| 6 | 97 | 9 | 10205.54 | 10184.97 | 787.597 | 7.72 | 164.34 | 15 |
| 7 | 6 | 1* | 10302.86 | 4051.53 | 2663.64 | 25.85 | ----- | ----- |
| 8 | 277 | 114 | 10298.47 | 10298.47 | 6322.67 | 61.39 | 318.29 | 27 |
| 9 | 145 | 45 | 10298.47 | 10298.47 | 7368.77 | 71.55 | 187.07 | 24 |
| 10 | 177 | 38 | 10302.86 | 10302.86 | 3450.46 | 33.49 | 253.81 | 32 |
| 11 | 103 | 11 | 10311.63 | 10311.63 | 3062.28 | 29.7 | 160.01 | 13 |
| 12 | 264 | 131 | 10324.81 | 10049.27 | 3318.62 | 32.14 | 316.55 | 50 |
| 13 | 92 | 51 | 10413.67 | 3152.55 | 1114.47 | 10.7 | 103.24 | 11 |
| 14 | 260 | 64 | 10413.67 | 9246.26 | 4271.76 | 41.02 | 309.22 | 22 |
| 15 | 414 | 83 | 10418.2 | 10418.2 | 6149.3 | 59.02 | 104.18 | 11 |
| 16 | 488 | 188 | 10427.28 | 10427.28 | 2715.42 | 26.04 | 624.67 | 95 |
| 17 | 155 | 35 | 10440.91 | 7916 | 1964.71 | 18.82 | 210.12 | 26 |
| 18 | 4 | 2* | 10525.68 | 4272.03 | 30.8304 | 0.29 | ----- | ----- |
| 19 | 166 | 47 | 10525.68 | 10525.68 | 587.138 | 5.58 | 212.71 | 23 |
| 20 | 384 | 89 | 10530.37 | 10530.37 | 4722.43 | 44.85 | 533.95 | 68 |
| 21 | 453 | 100 | 10539.75 | 10539.75 | 8364.88 | 79.37 | 580.81 | 55 |
| 22 | 362 | 74 | 10553.83 | 8924.7 | 4396.13 | 41.65 | 508.46 | 63 |
| 23 | 7 | 1* | 10634.48 | 9231.79 | 30.798 | 0.29 | ------ | ----- |
| 24 | 38 | 3* | 10634.48 | 10634.48 | 29.6136 | 0.28 | ------ | ----- |
| 25 | 285 | 83 | 10639.32 | 10639.32 | 805.378 | 7.57 | 355.3 | 35 |
| 26 | 517 | 157 | 10648.99 | 10648.99 | 5105.24 | 47.94 | 672.41 | 87 |
| 27 | 134 | 35 | 10663.53 | 10663.53 | 7241.23 | 67.91 | 182.52 | 25 |
| 28 | 77 | 14 | 10682.94 | 10440.1 | 4933.57 | 46.18 | 121.14 | 17 |
| 29 | 93 | 39 | 10745.02 | 8366.3 | 320.123 | 2.98 | 103.36 | 6 |
| 30 | 21 | 2* | 10740.04 | 10740.04 | 95.058 | 0.89 | ------ | ----- |
| 31 | 47 | 6 | 10740.04 | 10740.04 | 24.246 | 0.23 | 64.65 | 5 |
| 32 | 94 | 18 | 10745.02 | 10745.02 | 75.852 | 0.71 | 125.36 | 11 |
| 33 | 38 | 5 | 10755 | 10755 | 226.231 | 2.1 | 61.9 | 7 |
| 34 | 122 | 22 | 10769.98 | 10769.98 | 3209.76 | 29.8 | 182.2 | 23 |
| 35 | 240 | 144 | 10789.99 | 10789.99 | 6761.45 | 62.66 | 287.19 | 49 |
| 36 | 142 | 37 | 10815.06 | 3879.63 | 3012.78 | 27.86 | 185.82 | 22 |
| 37 | 43 | 20 | 10857.72 | 7935.91 | 316.573 | 2.92 | 49.62 | 5 |
| 38 | 34 | 14 | 10847.46 | 10847.46 | 1454.59 | 13.41 | 42.62 | 6 |
| 39 | 62 | 16 | 10842.33 | 10842.33 | 34.2972 | 0.32 | 80.09 | 8 |
| 40 | 43 | 6 | 10842.33 | 10842.33 | 315.392 | 2.91 | 60.41 | 5 |
| 41 | 134 | 17 | 10847.46 | 10847.46 | 458.489 | 4.23 | 280.54 | 40 |
| 42 | 123 | 14 | 10857.72 | 10857.72 | 563.494 | 5.19 | 198.82 | 19 |
| 43 | 17 | 2* | 10873.15 | 10873.15 | 253.112 | 2.33 | ------ | ----- |
| 44 | 64 | 24 | 10893.75 | 10893.75 | 593.957 | 5.45 | 82.8 | 13 |
| 45 | 106 | 87 | 10919.56 | 7205.38 | 2025.16 | 18.55 | 119.03 | 17 |
| 46 | 66 | 27 | 10950.62 | 1557.36 | 527.591 | 4.82 | 76.67 | 8 |
| 47 | 85 | 6 | 11760.73 | 6794.97 | 5830.71 | 49.58 | 24.03 | 22 |
| 48 | 490 | 32 | 11868.09 | 11663 | 10820.9 | 91.18 | 844.79 | 51 |
| 49 | 518 | 36 | 11982.28 | 11982.28 | 9976.04 | 83.26 | 849.08 | 48 |
| 50 | 229 | 20 | 12103.52 | 8150.96 | 6394.23 | 52.83 | 372.47 | 27 |
| 51 | 131 | 28 | 12232.01 | 1298.71 | 855.367 | 6.99 | 162.33 | 13 |
| 52 | 61 | 30 | 10994.19 | 9282.93 | 2348.2 | 21.36 | 73.81 | 11 |
| 53 | 64 | 13 | 10973.01 | 9612.65 | 3935.04 | 35.86 | 111.39 | 21 |
| 54 | 116 | 44 | 10957.15 | 10957.15 | 1877.64 | 17.14 | 165.5 | 37 |
| 55 | 66 | 43 | 10946.6 | 10946.6 | 379.721 | 3.47 | 74.88 | 10 |
| 56 | 62 | 19 | 10941.32 | 10941.32 | 172.75 | 1.58 | 81.99 | 12 |
| 57 | 76 | 43 | 10941.32 | 10941.32 | 1341.01 | 12.26 | 95.88 | 19 |
| 58 | 278 | 84 | 10946.6 | 10946.6 | 3065.16 | 28 | 360.82 | 49 |
| 59 | 156 | 35 | 10957.15 | 10957.15 | 292.514 | 2.67 | 197.67 | 16 |
| 60 | 26 | 3* | 10973.01 | 10973.01 | 222.901 | 2.03 | ------ | ----- |
| 61 | 19 | 3* | 10994.19 | 10994.19 | 161.392 | 1.47 | ------ | ----- |
| 62 | 28 | 8 | 11020.74 | 11020.74 | 163.3 | 1.48 | 57.57 | 19 |
| 63 | 43 | 9 | 11052.69 | 10740.2 | 481.082 | 4.35 | 93.02 | 22 |
| 64 | 50 | 20 | 11090.1 | 7745.58 | 670.932 | 6.05 | 61.42 | 8 |
| 65 | 95 | 25 | 11133.03 | 4766.92 | 573.638 | 5.15 | 118.62 | 12 |
| 66 | 167 | 58 | 11181.56 | 3067.92 | 774.961 | 6.93 | 213.98 | 33 |
| 67 | 8 | 1* | 11361.59 | 2.39 | 2.394 | 0.02 | ------ | ----- |
| 68 | 531 | 210 | 11433.43 | 8520.17 | 4712.63 | 41.22 | 648.22 | 83 |
| 69 | 73 | 24 | 11686.12 | 1469.21 | 1048.66 | 8.97 | 91.45 | 12 |
| 70 | 429 | 75 | 11783.25 | 10007.2 | 7954.37 | 67.51 | 620.72 | 69 |
| 71 | 512 | 67 | 11887.09 | 11887.09 | 11029.1 | 92.78 | 771.86 | 72 |
| 72 | 251 | 24 | 11997.84 | 11997.84 | 5949.67 | 49.59 | 346.74 | 20 |
| 73 | 344 | 100 | 12115.69 | 12019.5 | 5435.23 | 44.86 | 430.67 | 51 |
| 74 | 262 | 46 | 12240.86 | 8607.13 | 7215.72 | 58.95 | 382.81 | 43 |
| 75 | 95 | 54 | 11091.3 | 10079.6 | 3432.81 | 30.95 | 114.66 | 18 |
| 76 | 77 | 14 | 11069.54 | 11069.54 | 2221.62 | 20.07 | 117.31 | 15 |
| 77 | 165 | 38 | 11053.26 | 11053.26 | 1646.35 | 14.89 | 220.51 | 26 |
| 78 | 91 | 29 | 11042.41 | 11042.41 | 1088.79 | 9.86 | 111.17 | 11 |
| 79 | 159 | 99 | 11037 | 11037 | 1723.96 | 15.62 | 200.46 | 43 |
| 80 | 179 | 63 | 11037 | 11037 | 507.589 | 4.6 | 218.7 | 24 |
| 81 | 324 | 104 | 11042.41 | 11042.41 | 2065.05 | 18.7 | 402.57 | 48 |
| 82 | 286 | 80 | 11053.26 | 11053.26 | 4896.23 | 44.3 | 396.08 | 60 |
| 83 | 137 | 27 | 11069.54 | 11069.54 | 3224.41 | 29.13 | 165.6 | 10 |
| 84 | 86 | 33 | 11091.3 | 11091.3 | 991.134 | 8.94 | 97.27 | 8 |
| 85 | 69 | 17 | 11118.56 | 11118.56 | 175.957 | 1.58 | 100.52 | 16 |
| 86 | 128 | 39 | 11151.39 | 11151.39 | 199.318 | 1.79 | 180.84 | 32 |
| 87 | 28 | 6 | 11189.82 | 11189.82 | 210.787 | 1.88 | 58.29 | 14 |
| 88 | 10 | 1* | 11233.94 | 11233.94 | 170.237 | 1.52 | ------ | ----- |
| 89 | 14 | 2* | 11283.81 | 11222.4 | 381.035 | 3.38 | ------ | ----- |
| 90 | 623 | 167 | 11542.78 | 7332.33 | 2125.4 | 18.41 | 787.86 | 91 |
| 91 | 315 | 74 | 11622.96 | 8059.2 | 2485.58 | 21.39 | 401.77 | 39 |
| 92 | 171 | 41 | 11709.59 | 9401.84 | 2162.23 | 18.47 | 224.93 | 27 |
| 93 | 146 | 20 | 11802.82 | 9695.97 | 2316.33 | 19.63 | 206.69 | 17 |
| 94 | 248 | 47 | 11902.82 | 11707.3 | 3852.09 | 32.36 | 382.05 | 51 |
| 95 | 94 | 15 | 12009.78 | 12009.78 | 6629.36 | 55.2 | 138.86 | 15 |
| 96 | 78 | 9 | 12245.37 | 3500.8 | 2234.25 | 18.25 | 136.96 | 15 |
| 97 | 88 | 32 | 11185.03 | 4702.42 | 908.057 | 8.12 | 106.47 | 14 |
| 98 | 123 | 33 | 11162.71 | 11162.71 | 2003.07 | 17.94 | 173.69 | 30 |
| 99 | 175 | 44 | 11146.01 | 11146.01 | 1600.84 | 14.36 | 235.12 | 30 |
| 100 | 307 | 78 | 11134.89 | 11134.89 | 2426.19 | 21.79 | 411.19 | 55 |
| 101 | 145 | 22 | 11129.33 | 11129.33 | 1243.97 | 11.18 | 218.32 | 24 |
| 102 | 226 | 77 | 11129.33 | 11129.33 | 1748.63 | 15.71 | 288.01 | 36 |
| 103 | 191 | 31 | 11134.89 | 11134.89 | 3594.81 | 32.28 | 321.95 | 46 |
| 104 | 290 | 72 | 11146.01 | 11146.01 | 7770.34 | 69.71 | 359.71 | 37 |
| 105 | 115 | 32 | 11162.71 | 11162.71 | 1833.09 | 16.42 | 136.9 | 15 |
| 106 | 99 | 27 | 11185.03 | 11185.03 | 1133.62 | 10.14 | 129.25 | 16 |
| 107 | 133 | 38 | 11213.01 | 11213.01 | 759.964 | 6.78 | 166.33 | 19 |
| 108 | 94 | 18 | 11246.69 | 11246.69 | 852.437 | 7.58 | 124.53 | 13 |
| 109 | 77 | 17 | 11286.13 | 11286.13 | 258.944 | 2.29 | 140.28 | 31 |
| 110 | 77 | 13 | 11331.41 | 11331.41 | 33.5052 | 0.3 | 108.38 | 12 |
| 111 | 40 | 8 | 11382.6 | 11382.6 | 717.026 | 6.3 | 54.99 | 6 |
| 112 | 23 | 2* | 11439.81 | 11439.81 | 712.944 | 6.23 | ------ | ----- |
| 113 | 130 | 36 | 11503.12 | 11503.12 | 1014.32 | 8.82 | 167.99 | 22 |
| 114 | 82 | 26 | 11572.66 | 11572.66 | 1179.93 | 10.2 | 113.65 | 21 |
| 115 | 31 | 4* | 11648.56 | 5931.37 | 1103.74 | 9.48 | ------ | ----- |
| 116 | 76 | 25 | 11819.99 | 9636.11 | 5098.89 | 43.14 | 104.1 | 19 |
| 117 | 141 | 94 | 11915.84 | 9873.66 | 8850.13 | 74.27 | 156.45 | 14 |
| 118 | 122 | 34 | 12018.68 | 11435.7 | 10267.1 | 85.43 | 154.35 | 17 |
| 119 | 256 | 69 | 12128.72 | 12128.72 | 8724.27 | 71.93 | 362.45 | 53 |
| 120 | 32 | 28 | 12246.15 | 9971.4 | 5377.27 | 43.91 | 36.94 | 6 |
| 121 | 20 | 2* | 11252.5 | 11040.3 | 456.012 | 4.05 | ------ | ----- |
| 122 | 289 | 132 | 11235.38 | 11235.38 | 3691.61 | 32.86 | 345.87 | 45 |
| 123 | 364 | 181 | 11223.99 | 11223.99 | 5347.87 | 47.65 | 450.95 | 58 |
| 124 | 288 | 72 | 11218.3 | 11218.3 | 2877.48 | 25.65 | 390.29 | 47 |
| 125 | 263 | 48 | 11218.3 | 11218.3 | 3074.28 | 27.4 | 388.82 | 48 |
| 126 | 136 | 31 | 11223.99 | 11223.99 | 3300.51 | 29.41 | 189.21 | 26 |
| 127 | 14 | 2* | 11235.38 | 11235.38 | 4066.98 | 36.2 | ------ | ----- |
| 128 | 141 | 59 | 11252.5 | 11252.5 | 2733.44 | 24.29 | 161.93 | 19 |
| 129 | 186 | 134 | 11275.38 | 11275.38 | 4672.75 | 41.44 | 218.92 | 40 |
| 130 | 143 | 50 | 11304.05 | 11304.05 | 5062.47 | 44.78 | 165.52 | 16 |
| 131 | 123 | 67 | 11338.57 | 11338.57 | 3754.13 | 33.11 | 148.5 | 26 |
| 132 | 231 | 67 | 11379 | 11379 | 4310.19 | 37.88 | 304.58 | 39 |
| 133 | 303 | 161 | 11425.42 | 11425.42 | 5377.78 | 47.07 | 360.74 | 58 |
| 134 | 178 | 73 | 11477.91 | 11477.91 | 3464.53 | 30.18 | 229.49 | 40 |
| 135 | 237 | 114 | 11536.57 | 11536.57 | 4728.59 | 40.99 | 284.55 | 40 |
| 136 | 156 | 68 | 11601.51 | 11601.51 | 3057.17 | 26.35 | 179.98 | 20 |
| 137 | 161 | 109 | 11672.85 | 11672.85 | 3270.52 | 28.02 | 191.2 | 36 |
| 138 | 24 | 10 | 11750.73 | 4273.12 | 681.962 | 5.8 | 32.11 | 6 |
| 139 | 161 | 43 | 12130.77 | 10127.2 | 6607.23 | 54.47 | 202.45 | 21 |
| 140 | 76 | 99 | 12243.83 | 12136.9 | 7944.02 | 64.88 | 81.56 | 9 |
| 141 | 48 | 61 | 12364.53 | 5581.73 | 3626.55 | 29.33 | 57.21 | 19 |
| 142 | 14 | 11 | 11426.99 | 6506.78 | 1710.71 | 14.97 | 23.62 | 14 |
| 143 | 34 | 35 | 11391.65 | 11391.65 | 2697.2 | 23.68 | 46.87 | 24 |
| 144 | 6 | 12 | 11362.3 | 11362.3 | 1324.95 | 11.66 | 7.71 | 6 |
| 145 | 19 | 10 | 11338.89 | 11338.89 | 567.403 | 5 | 25.4 | 7 |
| 146 | 19 | 11 | 11321.36 | 11321.36 | 710.19 | 6.27 | 20.76 | 2 |
| 147 | 158 | 53 | 11309.7 | 11309.7 | 2091.84 | 18.5 | 190.04 | 19 |
| 148 | 216 | 78 | 11303.87 | 11303.87 | 2226.84 | 19.7 | 259.84 | 27 |
| 149 | 36 | 3* | 11303.87 | 11303.87 | 406.544 | 3.6 | ------ | ----- |
| 150 | 45 | 5 | 11309.7 | 11309.7 | 829.512 | 7.33 | 60.81 | 4 |
| 151 | 217 | 49 | 11321.36 | 11321.36 | 2308.51 | 20.39 | 257.84 | 16 |
| 152 | 291 | 89 | 11338.89 | 11338.89 | 3770.08 | 33.25 | 350.72 | 34 |
| 153 | 141 | 55 | 11362.3 | 11362.3 | 4919.58 | 43.3 | 173.32 | 24 |
| 154 | 254 | 91 | 11391.65 | 11391.65 | 4116.41 | 36.14 | 348.22 | 65 |
| 155 | 350 | 137 | 11426.99 | 11426.99 | 7225.32 | 63.23 | 413.83 | 46 |
| 156 | 229 | 40 | 11468.39 | 11468.39 | 8166.14 | 71.21 | 298.41 | 28 |
| 157 | 233 | 49 | 11515.92 | 11515.92 | 6664.04 | 57.87 | 294.45 | 29 |
| 158 | 256 | 151 | 11569.68 | 11569.68 | 5135.16 | 44.38 | 300.18 | 46 |
| 159 | 147 | 90 | 11629.77 | 11629.77 | 5184.11 | 44.58 | 176.11 | 33 |
| 160 | 114 | 47 | 11696.31 | 11696.31 | 1880.69 | 16.08 | 141.97 | 22 |
| 161 | 90 | 68 | 11769.41 | 11769.41 | 2228.58 | 18.94 | 101.47 | 13 |
| 162 | 26 | 6 | 11849.23 | 8664.93 | 1688.76 | 14.25 | 30.28 | 2 |
| 163 | 149 | 121 | 12130.61 | 7326.07 | 5369.29 | 44.26 | 182.56 | 45 |
| 164 | 205 | 65 | 12238.99 | 9291.41 | 7350.65 | 60.06 | 297.24 | 56 |
| 165 | 168 | 70 | 12355.03 | 4969.74 | 3819.79 | 30.92 | 225.99 | 46 |
| 166 | 13 | 13 | 11475.79 | 10730.2 | 575.122 | 5.01 | 21.59 | 17 |
| 167 | 3 | 9 | 11445.78 | 11445.78 | 719.622 | 6.29 | 3.26 | 1 |
| 168 | 9 | 5 | 11421.84 | 11421.84 | 1380.9 | 12.09 | 12.99 | 5 |
| 169 | 9 | 2* | 11403.92 | 11403.92 | 500.051 | 4.38 | ------ | ----- |
| 170 | 8 | 25 | 11392 | 11392 | 1284.6 | 11.28 | 8.96 | 4 |
| 171 | 250 | 33 | 11386.04 | 11386.04 | 2008 | 17.64 | 389.57 | 38 |
| 172 | 121 | 17 | 11386.04 | 11386.04 | 1292.29 | 11.35 | 159.89 | 11 |
| 173 | 268 | 39 | 11392 | 11392 | 3517.14 | 30.87 | 379.72 | 34 |
| 174 | 217 | 44 | 11403.92 | 11403.92 | 4467.24 | 39.17 | 251.85 | 12 |
| 175 | 335 | 87 | 11421.84 | 11421.84 | 5286.52 | 46.28 | 393.01 | 29 |
| 176 | 355 | 82 | 11475.79 | 11475.79 | 5819.91 | 50.71 | 495.67 | 68 |
| 177 | 106 | 105 | 11511.94 | 11511.94 | 5147.03 | 44.71 | 118.14 | 18 |
| 178 | 201 | 71 | 11554.28 | 11554.28 | 5362.83 | 46.41 | 246.01 | 29 |
| 179 | 221 | 58 | 11602.9 | 11602.9 | 5850.99 | 50.43 | 282.86 | 32 |
| 180 | 295 | 105 | 11657.9 | 11657.9 | 5178.05 | 44.42 | 399.68 | 73 |
| 181 | 163 | 60 | 11719.38 | 11719.38 | 6744.02 | 57.55 | 201.44 | 26 |
| 182 | 245 | 67 | 11787.47 | 11787.47 | 4338.59 | 36.81 | 319.58 | 38 |
| 183 | 51 | 37 | 11862.3 | 11862.3 | 3831.21 | 32.3 | 56.57 | 7 |
| 184 | 40 | 11 | 11944.02 | 11538.5 | 3293.28 | 27.57 | 46.99 | 3 |
| 185 | 31 | 5 | 12032.79 | 200.24 | 112.414 | 0.93 | 44.13 | 4 |
| 186 | 103 | 60 | 12343.3 | 5536.53 | 3541.45 | 28.69 | 132.05 | 29 |
| 187 | 30 | 13 | 12462.25 | 1483.66 | 958.77 | 7.69 | 44.82 | 15 |
| 188 | 17 | 7 | 11556.46 | 3544.78 | 487.228 | 4.22 | 25.46 | 7 |
| 189 | 63 | 24 | 11525.8 | 11272.6 | 2415.97 | 20.96 | 93.15 | 21 |
| 190 | 28 | 13 | 11501.34 | 11233.4 | 1584.18 | 13.77 | 36.87 | 7 |
| 191 | 14 | 6 | 11483.04 | 9264.4 | 515.768 | 4.49 | 19.57 | 5 |
| 192 | 32 | 22 | 11470.86 | 11470.86 | 3118.28 | 27.18 | 114.71 | 29 |
| 193 | 98 | 13 | 11464.78 | 11464.78 | 2584.51 | 22.54 | 146.91 | 14 |
| 194 | 268 | 49 | 11464.78 | 11464.78 | 3452 | 30.11 | 339.01 | 27 |
| 195 | 255 | 50 | 11470.86 | 11470.86 | 4036.34 | 35.19 | 294.98 | 16 |
| 196 | 189 | 110 | 11483.04 | 11483.04 | 4443.9 | 38.7 | 215.27 | 27 |
| 197 | 191 | 75 | 11501.34 | 11501.34 | 3582.53 | 31.15 | 216.63 | 18 |
| 198 | 189 | 126 | 11525.8 | 11525.8 | 4068.77 | 35.3 | 210.34 | 21 |
| 199 | 158 | 170 | 11556.46 | 11556.46 | 4954.29 | 42.87 | 174.02 | 27 |
| 200 | 58 | 20 | 11593.38 | 11593.38 | 1034.87 | 8.93 | 69.25 | 7 |
| 201 | 145 | 88 | 11636.64 | 11636.64 | 2500.77 | 21.49 | 161.85 | 17 |
| 202 | 258 | 70 | 11686.32 | 11686.32 | 3510.01 | 30.04 | 354.37 | 54 |
| 203 | 283 | 236 | 11742.53 | 11742.53 | 6943.98 | 59.14 | 331.42 | 69 |
| 204 | 198 | 48 | 11805.37 | 11805.37 | 6966.49 | 59.01 | 278.86 | 43 |
| 205 | 396 | 208 | 11874.97 | 11352.7 | 5301.02 | 44.64 | 482.09 | 83 |
| 206 | 40 | 73 | 12035.05 | 5669.84 | 1972.92 | 16.39 | 41.79 | 4 |
| 207 | 17 | 1* | 12125.85 | 392.63 | 196.279 | 1.62 | ------ | ----- |
| 208 | 18 | 9 | 11602.32 | 1770.73 | 171.587 | 1.48 | 21.31 | 3 |
| 209 | 31 | 8 | 11558.7 | 2349.17 | 293.076 | 2.54 | 38.16 | 3 |
| 210 | 132 | 78 | 11546.28 | 11546.28 | 4193.45 | 36.32 | 165.76 | 38 |
| 211 | 78 | 10 | 11540.07 | 11540.07 | 2214.19 | 19.19 | 98.15 | 5 |
| 212 | 124 | 36 | 11540.07 | 11540.07 | 1557.65 | 13.5 | 161.45 | 21 |
| 213 | 116 | 36 | 11546.28 | 11546.28 | 1009.76 | 8.75 | 143.17 | 19 |
| 214 | 102 | 28 | 11558.7 | 11558.7 | 1280.07 | 11.07 | 137.89 | 21 |
| 215 | 131 | 76 | 11577.37 | 11577.37 | 1718.73 | 14.85 | 150.51 | 20 |
| 216 | 144 | 196 | 11602.32 | 11602.32 | 4030.19 | 34.74 | 167.24 | 49 |
| 217 | 140 | 78 | 11633.61 | 11633.61 | 6505.7 | 55.92 | 164.11 | 23 |
| 218 | 181 | 58 | 11671.29 | 11671.29 | 4400.49 | 37.7 | 209.93 | 16 |
| 219 | 251 | 97 | 11715.44 | 11715.44 | 5726.85 | 48.88 | 322.1 | 52 |
| 220 | 394 | 146 | 11766.15 | 11766.15 | 4473.19 | 38.02 | 503.38 | 75 |
| 221 | 462 | 199 | 11823.53 | 11823.53 | 7590.63 | 64.2 | 539.97 | 60 |
| 222 | 272 | 105 | 11887.69 | 11887.69 | 3956.61 | 33.28 | 368.34 | 68 |
| 223 | 160 | 35 | 11958.76 | 9266.68 | 1887.17 | 15.78 | 228.04 | 30 |
| 224 | 53 | 12 | 11630.88 | 2989.92 | 965.516 | 8.3 | 65.95 | 6 |
| 225 | 162 | 26 | 11618.22 | 11618.22 | 4791.37 | 41.24 | 218.42 | 18 |
| 226 | 84 | 5 | 11611.89 | 11611.89 | 1519.41 | 13.08 | 146.57 | 9 |
| 227 | 128 | 23 | 11611.89 | 11611.89 | 662.13 | 5.7 | 170.1 | 16 |
| 228 | 114 | 25 | 11618.22 | 11618.22 | 365.407 | 3.15 | 136.92 | 12 |
| 229 | 98 | 55 | 11630.88 | 11630.88 | 1715.61 | 14.75 | 108.51 | 9 |
| 230 | 73 | 47 | 11649.91 | 11649.91 | 4551.96 | 39.07 | 85.57 | 13 |
| 231 | 110 | 50 | 11675.34 | 11675.34 | 5077.7 | 43.49 | 128.48 | 15 |
| 232 | 203 | 49 | 11745.64 | 11745.64 | 6503.88 | 55.37 | 259.28 | 26 |
| 233 | 354 | 192 | 11842.36 | 11842.36 | 8364.86 | 70.64 | 445.43 | 88 |
| 234 | 414 | 208 | 11900.87 | 11249.3 | 6571.22 | 55.22 | 502.21 | 81 |
| 235 | 219 | 31 | 11966.31 | 4381.68 | 1374.64 | 11.49 | 323.03 | 31 |
| 236 | 55 | 5 | 12038.81 | 494.95 | 151.034 | 1.25 | 120.39 | 10 |
| 237 | 40 | 4* | 11699.56 | 1105.12 | 352.818 | 3.02 | ------ | ----- |
| 238 | 126 | 39 | 11686.67 | 11675.5 | 5382.5 | 46.06 | 172.7 | 28 |
| 239 | 71 | 9 | 11680.23 | 11680.23 | 1371.21 | 11.74 | 99.22 | 4 |
| 240 | 109 | 23 | 11680.23 | 11680.23 | 1051.07 | 9 | 134.96 | 12 |
| 241 | 91 | 12 | 11686.67 | 11686.67 | 634.378 | 5.43 | 149.82 | 17 |
| 242 | 67 | 5 | 11699.56 | 11699.56 | 977.918 | 8.36 | 101.93 | 6 |
| 243 | 40 | 15 | 11718.94 | 11718.94 | 2523.81 | 21.54 | 48.49 | 6 |
| 244 | 236 | 66 | 11744.84 | 11744.84 | 2409.07 | 20.51 | 313.28 | 46 |
| 245 | 308 | 104 | 11777.31 | 11777.31 | 8745.18 | 74.25 | 404.76 | 65 |
| 246 | 276 | 55 | 11816.42 | 11816.42 | 5678.04 | 48.05 | 362.29 | 35 |
| 247 | 316 | 52 | 11862.27 | 11862.27 | 6232.17 | 52.54 | 471.6 | 50 |
| 248 | 110 | 54 | 11914.94 | 11223.1 | 3921.08 | 32.91 | 128.92 | 16 |
| 249 | 211 | 38 | 11974.54 | 3479.66 | 1460.3 | 12.2 | 303.44 | 37 |
| 250 | 82 | 11 | 11751.61 | 9714.65 | 5315.47 | 45.23 | 116.27 | 11 |
| 251 | 48 | 8 | 11745.06 | 11745.06 | 536.832 | 4.57 | 81.28 | 13 |
| 252 | 10 | 1* | 11751.61 | 11751.61 | 378.436 | 3.22 | ------ | ----- |
| 253 | 60 | 9 | 11764.73 | 11764.73 | 1075.81 | 9.14 | 123 | 21 |
| 254 | 157 | 20 | 11784.44 | 11784.44 | 1403.37 | 11.91 | 349.09 | 55 |
| 255 | 42 | 6 | 11810.78 | 11810.78 | 487.958 | 4.13 | 98.13 | 19 |
| 256 | 168 | 105 | 11843.81 | 11843.81 | 4446.45 | 37.54 | 195.9 | 31 |
| 257 | 208 | 125 | 11883.61 | 11883.61 | 6832.94 | 57.5 | 240.77 | 33 |
| 258 | 254 | 75 | 11930.25 | 9523.24 | 4428.18 | 37.12 | 337.79 | 48 |
| 259 | 29 | 4* | 11983.84 | 2041.11 | 781.207 | 6.52 | ------ | ----- |
| 260 | 137 | 36 | 11813.04 | 7471.75 | 3852.64 | 32.61 | 166.5 | 15 |
| 261 | 86 | 11 | 11806.38 | 11806.38 | 1573.08 | 13.32 | 118.06 | 5 |
| 262 | 53 | 8 | 11806.38 | 11806.38 | 1381.42 | 11.7 | 81.72 | 9 |
| 263 | 46 | 8 | 11813.04 | 11813.04 | 908.971 | 7.69 | 62.91 | 7 |
| 264 | 151 | 17 | 11826.36 | 11826.36 | 897.455 | 7.59 | 279.38 | 33 |
| 265 | 266 | 87 | 11846.39 | 11846.39 | 3019.91 | 25.49 | 310.72 | 29 |
| 266 | 304 | 65 | 11873.16 | 11873.16 | 3086.35 | 25.99 | 423.12 | 48 |
| 267 | 67 | 14 | 11906.73 | 11906.73 | 1134.44 | 9.53 | 101.61 | 16 |
| 268 | 82 | 11 | 11870.92 | 2555.74 | 1107.13 | 9.33 | 161.99 | 23 |
| 269 | 428 | 154 | 11864.17 | 11862 | 6493.08 | 54.73 | 500.61 | 52 |
| 270 | 105 | 8 | 11864.17 | 11864.17 | 1186.84 | 10 | 160.78 | 11 |
| 271 | 30 | 1* | 11870.92 | 11870.92 | 1304.65 | 10.99 | ------ | ----- |
| 272 | 31 | 3* | 11884.45 | 11884.45 | 847.476 | 7.13 | ------ | ----- |
| 273 | 474 | 153 | 11904.78 | 11904.78 | 4272.56 | 35.89 | 580.92 | 60 |
| 274 | 351 | 49 | 11931.95 | 11931.95 | 2998.82 | 25.13 | 621.51 | 80 |
| 275 | 308 | 64 | 11918.41 | 8071.17 | 6299.34 | 52.85 | 400.37 | 42 |
| 276 | 137 | 35 | 11918.41 | 11918.41 | 2860.98 | 24 | 157.21 | 9 |
| 277 | 99 | 25 | 11925.26 | 11925.26 | 1861.93 | 15.61 | 142.41 | 25 |
| 278 | 100 | 20 | 11938.98 | 11938.98 | 1420.8 | 11.9 | 131.47 | 15 |
| 279 | 345 | 77 | 11959.59 | 11959.59 | 3222.73 | 26.95 | 434.58 | 41 |
| 280 | 402 | 87 | 11987.15 | 11987.15 | 4823.68 | 40.24 | 550.06 | 64 |
| 281 | 172 | 21 | 11969.09 | 3777.16 | 1747.35 | 14.6 | 239.24 | 19 |
| 282 | 435 | 145 | 11969.09 | 11969.09 | 7228.99 | 60.4 | 502.39 | 55 |
| 283 | 132 | 37 | 11976.03 | 11976.03 | 3186.86 | 26.61 | 155.04 | 14 |
| 284 | 73 | 11 | 11989.93 | 11989.93 | 2891.04 | 24.11 | 99.86 | 9 |
| 285 | 175 | 24 | 12010.82 | 12010.82 | 4136.81 | 34.44 | 368.1 | 57 |
| 286 | 449 | 105 | 12038.74 | 12038.74 | 5072.48 | 42.13 | 590.2 | 65 |
| 287 | 238 | 50 | 12016.2 | 11394.3 | 6126.91 | 50.99 | 295.7 | 26 |
| 288 | 220 | 26 | 12023.23 | 12023.23 | 1721.46 | 14.32 | 120.23 | 14 |
| 289 | 460 | 132 | 12037.3 | 12037.3 | 5132.68 | 42.64 | 585.7 | 77 |
| 290 | 383 | 96 | 12058.44 | 12058.44 | 4500.24 | 37.32 | 500.9 | 61 |
| 291 | 74 | 10 | 12086.7 | 11862 | 1298.32 | 10.74 | 171.63 | 29 |
| 292 | 108 | 14 | 12059.73 | 4605.15 | 1676.22 | 13.9 | 160.72 | 16 |
| 293 | 547 | 113 | 12066.84 | 12066.84 | 7699.41 | 63.81 | 743.91 | 83 |
| 294 | 389 | 66 | 12081.06 | 12081.06 | 3878.13 | 32.1 | 524.7 | 49 |
| 295 | 609 | 242 | 12102.44 | 12102.44 | 3810.19 | 31.48 | 795.55 | 14 |
| 296 | 566 | 117 | 12106.85 | 11228 | 3830.05 | 31.64 | 801.95 | 102 |
| 297 | 581 | 82 | 12121.22 | 12121.22 | 3721.2 | 30.7 | 840.69 | 80 |
| 298 | 129 | 12 | 12142.81 | 12142.81 | 1452.12 | 11.96 | 186.5 | 12 |
| 299 | 110 | 11 | 12143.25 | 7926.2 | 1774.56 | 14.61 | 197.13 | 21 |
| 300 | 183 | 24 | 12157.76 | 12157.76 | 5152.9 | 42.38 | 337.46 | 44 |
| 301 | 377 | 58 | 12190.66 | 9771.4 | 3191.54 | 26.18 | 741.55 | 138 |

*depicts that the grids having <5 sampling plots were not included in fitting the clench function

(207-grids were fully covered and 94-grids were partially covered within Indian geographic area)
