# Supplementary material for: Optimized grid representation of plant species richness in India—Utility of an existing national database in integrated ecological analysis
Source: PLoS One. 2017 Mar 15;12(3):e0173774. doi: 10.1371/journal.pone.0173774 (PMC5352167; doi:10.1371/journal.pone.0173774)
Supplement: S3 Table — (DOCX) [file pone.0173774.s005.docx]

**S3 Table. Table showing the observed and asymptote species richness of India at 2˚ scale**

| **S. no.** | **Observed SR** | **Plot** | **Total grid area (km^2^)** | **Indian geo. Area (km^2^)** | **Indian veg area (km^2^)** | **Total grid veg area (%)** | **Asymptote (a/b) SR** | **Expected effort to 70% completeness** (N70%) |
| --- | --- | --- | --- | --- | --- | --- | --- | --- |
| 1 | 18 | 1* | 40502 | 9239 | 5282.67 | 13.04 | ----- | ----- |
| 2 | 316 | 163 | 40476 | 40253 | 20259.09 | 50.05 | 375.16 | 48 |
| 3 | 347 | 116 | 40471 | 35951 | 8998.34 | 22.23 | 440.49 | 59 |
| 4 | 97 | 9 | 40654 | 33839 | 1363.53 | 3.35 | 164.34 | 14 |
| 5 | 6 | 1* | 41425 | 4094 | 2675.55 | 6.46 | ----- | ----- |
| 6 | 507 | 274 | 41463 | 33037 | 19270.67 | 46.48 | 569.69 | 54 |
| 7 | 853 | 320 | 41457 | 41457 | 15353.03 | 37.03 | 1140.74 | 205 |
| 8 | 354 | 166 | 41562 | 22039 | 8190.06 | 19.71 | 434.09 | 72 |
| 9 | 195 | 53 | 42279 | 34649 | 678.13 | 1.60 | 240.65 | 22 |
| 10 | 995 | 429 | 42356 | 42356 | 19022.83 | 44.91 | 1233.55 | 182 |
| 11 | 493 | 123 | 42439 | 31384 | 16594.09 | 39.10 | 668.49 | 86 |
| 12 | 117 | 73 | 43177 | 27204 | 2091.16 | 4.84 | 129.26 | 11 |
| 13 | 114 | 30 | 43162 | 43162 | 467.23 | 1.08 | 148.47 | 17 |
| 14 | 243 | 54 | 43203 | 43203 | 1322.18 | 3.06 | 331.55 | 40 |
| 15 | 295 | 192 | 43325 | 43325 | 10843.25 | 25.03 | 354.52 | 67 |
| 16 | 248 | 151 | 43513 | 12629 | 5569.62 | 12.80 | 333.47 | 113 |
| 17 | 85 | 6 | 46658 | 7482 | 6135.46 | 13.15 | 222.63 | 22 |
| 18 | 809 | 68 | 47425 | 27859 | 25017.65 | 52.75 | 1314.63 | 91 |
| 19 | 323 | 48 | 48391 | 10729 | 8108.67 | 16.76 | 431.98 | 31 |
| 20 | 145 | 111 | 44104 | 40042 | 11924.44 | 27.04 | 167.68 | 27 |
| 21 | 231 | 154 | 43997 | 43997 | 4987.95 | 11.34 | 263.17 | 35 |
| 22 | 255 | 224 | 43955 | 43955 | 3742.77 | 8.52 | 296.11 | 55 |
| 23 | 506 | 303 | 43997 | 43997 | 10291.03 | 23.39 | 607.01 | 105 |
| 24 | 197 | 66 | 44126 | 44126 | 4608.10 | 10.44 | 226.04 | 16 |
| 25 | 159 | 73 | 44319 | 44012 | 1477.25 | 3.33 | 224.56 | 71 |
| 26 | 123 | 52 | 44655 | 34912 | 1625.45 | 3.64 | 144.69 | 16 |
| 27 | 168 | 60 | 45043 | 22936 | 1673.12 | 3.71 | 213.91 | 33 |
| 28 | 8 | 1* | 45534 | 11102 | 510.36 | 1.12 | ----- | ----- |
| 29 | 895 | 451 | 46101 | 23910 | 10694.62 | 23.20 | 1074.44 | 158 |
| 30 | 280 | 85 | 46803 | 20523 | 5591.55 | 11.95 | 350.54 | 40 |
| 31 | 838 | 204 | 47501 | 45525 | 29959.01 | 63.07 | 1087.44 | 119 |
| 32 | 503 | 133 | 48564 | 39749 | 22499.11 | 46.33 | 603.19 | 52 |
| 33 | 262 | 46 | 49415 | 9683 | 8504.27 | 17.21 | 382.81 | 43 |
| 34 | 144 | 67 | 44870 | 30390 | 3389.60 | 7.55 | 172.36 | 26 |
| 35 | 507 | 435 | 44738 | 44738 | 13059.89 | 29.19 | 577.40 | 92 |
| 36 | 449 | 219 | 44693 | 44693 | 8940.33 | 20.00 | 563.69 | 97 |
| 37 | 368 | 136 | 44738 | 44738 | 18749.20 | 41.91 | 442.08 | 55 |
| 38 | 296 | 252 | 44874 | 44874 | 10367.20 | 23.10 | 352.00 | 83 |
| 39 | 243 | 173 | 45100 | 45100 | 10410.90 | 23.08 | 278.49 | 42 |
| 40 | 416 | 258 | 45420 | 45420 | 9961.83 | 21.93 | 497.98 | 93 |
| 41 | 325 | 197 | 45835 | 45835 | 9594.93 | 20.93 | 396.08 | 75 |
| 42 | 284 | 239 | 46348 | 46348 | 8621.85 | 18.60 | 337.05 | 74 |
| 43 | 41 | 14 | 46987 | 11528 | 2597.35 | 5.53 | 65.01 | 15 |
| 44 | 184 | 119 | 47732 | 20765 | 19300.97 | 40.44 | 209.32 | 25 |
| 45 | 442 | 245 | 48523 | 45811 | 38706.29 | 79.77 | 515.80 | 62 |
| 46 | 55 | 89 | 49443 | 15801 | 13059.19 | 26.41 | 62.43 | 18 |
| 47 | 50 | 59 | 45795 | 23955 | 4814.60 | 10.51 | 66.51 | 33 |
| 48 | 34 | 36 | 45577 | 42790 | 3926.25 | 8.61 | 40.93 | 12 |
| 49 | 171 | 91 | 45460 | 44219 | 4516.62 | 9.94 | 194.40 | 20 |
| 50 | 409 | 131 | 45378 | 45378 | 5933.63 | 13.08 | 499.12 | 50 |
| 51 | 411 | 137 | 45425 | 45425 | 11113.91 | 24.47 | 494.05 | 48 |
| 52 | 499 | 231 | 45567 | 45567 | 17834.90 | 39.14 | 574.95 | 62 |
| 53 | 600 | 415 | 45805 | 45805 | 22309.10 | 48.70 | 696.70 | 115 |
| 54 | 433 | 218 | 46140 | 46140 | 26047.92 | 56.45 | 529.53 | 92 |
| 55 | 470 | 406 | 46575 | 46575 | 22241.89 | 47.76 | 545.00 | 109 |
| 56 | 315 | 219 | 47114 | 47114 | 12274.53 | 26.05 | 355.94 | 42 |
| 57 | 68 | 22 | 47759 | 20453 | 5098.57 | 10.68 | 78.94 | 6 |
| 58 | 149 | 121 | 48500 | 7372 | 5909.90 | 12.19 | 182.56 | 45 |
| 59 | 262 | 208 | 49391 | 21297 | 19812.64 | 40.11 | 320.79 | 78 |
| 60 | 17 | 7 | 46411 | 3561 | 486.85 | 1.05 | 25.46 | 7 |
| 61 | 102 | 46 | 46209 | 25455 | 4257.82 | 9.21 | 133.17 | 25 |
| 62 | 164 | 114 | 46058 | 31083 | 7972.38 | 17.31 | 204.96 | 51 |
| 63 | 358 | 108 | 46008 | 46008 | 9810.96 | 21.32 | 433.47 | 43 |
| 64 | 344 | 224 | 46058 | 46058 | 10782.06 | 23.41 | 370.27 | 29 |
| 65 | 285 | 473 | 46205 | 46205 | 13398.08 | 29.00 | 304.80 | 43 |
| 66 | 263 | 326 | 46453 | 46453 | 16887.72 | 36.35 | 279.51 | 27 |
| 67 | 570 | 401 | 46803 | 46803 | 16195.82 | 34.60 | 673.86 | 123 |
| 68 | 654 | 588 | 47258 | 47258 | 25467.85 | 53.89 | 742.62 | 119 |
| 69 | 443 | 243 | 47862 | 25603 | 8379.25 | 17.51 | 550.39 | 109 |
| 70 | 40 | 74 | 48440 | 6069 | 2171.33 | 4.48 | 41.77 | 4 |
| 71 | 225 | 81 | 46634 | 27428 | 11495.37 | 24.65 | 274.44 | 32 |
| 72 | 194 | 60 | 46583 | 46583 | 4607.39 | 9.89 | 230.68 | 21 |
| 73 | 169 | 97 | 46634 | 46634 | 3695.61 | 7.92 | 193.92 | 22 |
| 74 | 295 | 178 | 46788 | 46788 | 14561.04 | 31.12 | 346.29 | 50 |
| 75 | 502 | 208 | 47045 | 47045 | 30548.22 | 64.93 | 646.27 | 113 |
| 76 | 532 | 298 | 47440 | 46743 | 22673.21 | 47.79 | 636.52 | 98 |
| 77 | 498 | 277 | 47878 | 19162 | 9435.33 | 19.71 | 621.55 | 121 |
| 78 | 55 | 5 | 48437 | 496 | 151.29 | 0.31 | 74.68 | 4 |
| 79 | 165 | 47 | 47145 | 17218 | 9174.62 | 19.46 | 201.29 | 19 |
| 80 | 164 | 27 | 47102 | 47102 | 4805.86 | 10.20 | 208.95 | 16 |
| 81 | 190 | 35 | 47155 | 47155 | 3260.73 | 6.91 | 271.35 | 33 |
| 82 | 472 | 178 | 47314 | 47314 | 7980.49 | 16.87 | 587.80 | 82 |
| 83 | 314 | 244 | 47617 | 44299 | 12759.82 | 26.80 | 369.42 | 71 |
| 84 | 261 | 79 | 47927 | 13489 | 5408.74 | 11.29 | 296.99 | 67 |
| 85 | 82 | 11 | 47596 | 2570 | 1108.75 | 2.33 | 161.99 | 22 |
| 86 | 558 | 261 | 47585 | 43746 | 16848.19 | 35.41 | 646.21 | 74 |
| 87 | 196 | 49 | 47619 | 47619 | 5429.79 | 11.40 | 241.17 | 27 |
| 88 | 666 | 366 | 47783 | 47783 | 15329.00 | 32.08 | 782.92 | 106 |
| 89 | 471 | 216 | 47971 | 27936 | 15289.81 | 31.87 | 554.82 | 72 |
| 90 | 573 | 206 | 48026 | 48026 | 12924.62 | 26.91 | 680.66 | 73 |
| 91 | 668 | 235 | 48204 | 47977 | 15012.41 | 31.14 | 834.98 | 111 |
| 92 | 108 | 14 | 48302 | 5163 | 1689.34 | 3.50 | 160.72 | 15 |
| 93 | 1244 | 378 | 48381 | 47507 | 19213.67 | 39.71 | 1639.73 | 238 |
| 94 | 631 | 254 | 48559 | 42354 | 6904.93 | 14.22 | 771.70 | 109 |
| 95 | 474 | 93 | 48663 | 31814 | 10262.77 | 21.09 | 705.69 | 94 |

*depicts that the grids having <5 sampling plots were not included in fitting the clench function

(40-grids were fully covered and 55-grids were partially covered within Indian geographic area)

Please note that all the insufficiently sampled grids at 2^0^ would reveal insufficiency at 1^0^ grids too.
